# Glutamate and GABA as rapid effectors of hypothalamic “peptidergic” neurons

**DOI:** 10.3389/fnbeh.2012.00081

**Published:** 2012-11-26

**Authors:** Cornelia Schöne, Denis Burdakov

**Affiliations:** ^1^MRC National Institute for Medical ResearchLondon, UK; ^2^King's College LondonLondon, UK

**Keywords:** hypothalamus, appetite, sleep, hypocretin, orexin, glutamate, neuropeptide

## Abstract

Vital hypothalamic neurons regulating hunger, wakefulness, reward-seeking, and body weight are often defined by unique expression of hypothalamus-specific neuropeptides. Gene-ablation studies show that some of these peptides, notably orexin/hypocretin (hcrt/orx), are themselves critical for stable states of consciousness and metabolic health. However, neuron-ablation studies often reveal more severe phenotypes, suggesting key roles for co-expressed transmitters. Indeed, most hypothalamic neurons, including hcrt/orx cells, contain fast transmitters glutamate and GABA, as well as several neuropeptides. What are the roles and relations between different transmitters expressed by the same neuron? Here, we consider signaling codes for releasing different transmitters in relation to transmitter and receptor diversity in behaviorally defined, widely projecting “peptidergic” neurons, such as hcrt/orx cells. We then discuss latest optogenetic studies of endogenous transmitter release from defined sets of axons *in situ*, which suggest that recently characterized vital peptidergic neurons [e.g., hcrt/orx, proopiomelanocortin (POMC), and agouti-related peptide (AgRP) cells], as well as classical modulatory neurons (e.g., dopamine and acetylcholine cells), all use fast transmitters to control their postsynaptic targets. These optogenetic insights are complemented by recent observations of behavioral deficiencies caused by genetic ablation of fast transmission from specific neuropeptidergic and aminergic neurons. Powerful and fast (millisecond-scale) GABAergic and glutamatergic signaling from neurons previously considered to be primarily “modulatory” raises new questions about the roles of slower co-transmitters they co-express.

## Co-transmission and widely projecting neurons coordinating vital brain functions

Although neurons are often classified based on a neurotransmitter they contain (e.g., “cholinergic” or “GABAergic” cells), most if not all neurons contain—and use—more than one neurotransmitter. Yet, the relative roles of these “co-transmitters,” or even whether they are actually released together, are unclear in even the oldest and most fundamental processes, such as sleep and feeding behavior. An interesting example is offered by recent studies of hypothalamic neurons that co-express neuropeptide Y (NPY) and agouti-related peptide (AgRP), and whose electrical activity drives feeding (Aponte et al., 2011). Destruction of these cells leads to starvation (Gropp et al., 2005; Luquet et al., 2005), which has been attributed to loss of the neuropeptides that they contain. However, while NPY and AgRP cause feeding and weight gain when artificially infused into the brain, and their expression is regulated by body energy status, the weight loss seen when NPY/AgRP cells are destroyed is recapitulated by targeted deletion of their ability to release GABA, rather than NPY or AgRP (Tong et al., 2008). Are NPY and AgRP, then, relatively minor modulators of energy balance, while GABA is the “main” transmitter used by NPY/AgRP cells to drive feeding and weight gain? This does not appear to be the case, since when NPY/AgRP cell terminals are selectively stimulated using optogenetics at a behaviorally crucial projection site, the resulting feeding response can be strongly inhibited by antagonists of either GABA or NPY receptors (Atasoy et al., 2012). This illustrates that, in terms of translating the electrical activity in the same set of axons to behavior, neuropeptides and small molecule transmitters are not interchangeable and can be equally essential. How they co-operate is a key unresolved question.

The last decade brought about a revolution in our understanding of hypothalamic neurotransmitters underlying states of consciousness and metabolic health. Several hypothalamus-specific neuropeptides have been shown to be essential for normal sleep-wake cycles and body weight. For example, the knockout of hypocretin/orexin (hcrt/orx) produces narcolepsy (Chemelli et al., 1999), making hcrt/orx the first peptide messenger known to be essential for normal wakefulness and for environment-appropriate switches in the states of consciousness in mammals (De Lecea et al., 2006; Sakurai, 2007). Mutations in neuropeptide precursors, such as proopiomelanocortin (POMC), have been linked to obesity (Farooqi and O'Rahilly, 2006). Selective ablation of small-molecule-mediated neurotransmission in genetically defined populations of neurons demonstrated that glutamate release from ventromedial hypothalamic neurons is critical for preventing hypoglycemia (Tong et al., 2007), while GABA release from leptin receptor neurons is critical for the anti-obesity effects of leptin in the brain (Vong et al., 2011). Such important genetic, pharmacological, and anatomical studies identify cells and transmitters maintaining key aspects of health, but not how they do it. For example, recent optogenetic work highlighted that different firing rates in the same population of hypothalamic neuropeptidergic neurons can produce strikingly different behaviors (Adamantidis et al., 2007; Aponte et al., 2011). How does this link with the above-mentioned molecular knowledge? Many hypothalamic neurons regulating alertness and metabolic health are widely projecting and contain several neurotransmitters, but can it be assumed that they release all their transmitters at all projection targets every time an action potential is fired? This seems irrational, for example, because these molecules may issue “contradictory orders” (e.g., excitatory hcrt/orx and inhibitory dynorphin co-expressed in hcrt/orx cells, Chou et al., 2001). Furthermore, evidence from other systems indicates that this assumption is not safe. In contradiction to a version of the “Dale's principle” proposing that a neuron uses the same transmitters at all its synapses, multiple neuropeptides derived from a common precursor can be differentially packaged and transported to different neuronal processes, suggesting that neurons do not always release the same set of chemical messengers from all their endings (Fisher et al., 1988; Sossin et al., 1990). To add further complexity, the same stimulus can have differential effect on the release of the same neuropeptide from different parts of the same neuron (Sabatier et al., 2003).

The language of the brain is both chemical and electrical, and to add the “how” dimension to the “what/where” knowledge supplied by recent breakthroughs, causal links need to be made between electrical activity and transmitter release at physiologically defined sites. The aim of this review is to provide a wide-ranging overview of key concepts relevant to such a broader understanding, with selected examples from many fields of neuroscience, but with later emphasis on hypothalamic hcrt/orx cells. We discuss complementary properties of different transmitters and electrical codes for their release. Then, taking the hcrt/orx neurons as an example, we consider what their neurochemical and electrophysiological diversity may achieve. Finally, we discuss recent insights from optogenetics—currently the only technique that allows causal links to be made between axonal electrical activity and neurotransmitter release. This review is not intended to be exhaustive or comprehensive, and broader aspects of neuropeptide transmission are covered in more detail in other recent reviews (Van Den Pol, 2012). Our aim is to integrate older concepts with new type of knowledge emerging from modern genetic and optogenetic deconstruction of neural circuits. This is likely to be relevant to some of the most frequent medical problems of today, such as disorders of sleep and energy balance (Leger and Bayon, 2010; Lam and Leroith, 2012).

## Why have more than two transmitters?

Neuronal processing replies on co-operation of intrinsic electrical activity generated by neurons independently of any input, and the input signals they receive from one another (neurotransmitters) and from other cells (hormones). Since synaptic inputs essentially provide ON/OFF signals, one may ask why it is necessary to spend energy on making more than two neurotransmitters, one excitatory and one inhibitory. Indeed, early evidence from hypothalamic circuits shows that most synaptic activity is eliminated by blocking glutamate and GABA receptors (Van Den Pol et al., 1990; Van Den Pol, 2003). Why does the hypothalamus, and other brain regions, contain dozens of peptide transmitters in addition to glutamate and GABA, especially considering that most, if not all, of these peptidergic neurons are also capable of releasing glutamate and/or GABA (Atasoy et al., 2008, 2012; Dicken et al., 2012; Schöne et al., 2012)? As illustrated by clear behavioral phenotypes of knockouts of hypothalamic neuropeptides such as hcrt/orx (Chemelli et al., 1999) and melanin-concentrating hormone (Shimada et al., 1998), peptides are clearly critical for vital brain functions. Yet, it is unclear why neural processing requires the many cellular actions of exogenously applied neuropeptides that have been identified to-date (Van Den Pol, 2012).

What properties of neuropeptides add behaviorally essential dimensions to signaling within neural circuits, and why cannot these dimensions be provided by fast transmitters? An often-used argument is that neuropeptides are more capable of prolonged actions, due to their slower breakdown and higher receptor affinity (Salio et al., 2006). However, this property is not fundamentally different from fast transmitters, which are also capable of eliciting prolonged effects since, like neuropeptides, they can affect neuronal activity long after their release, by binding to slow-acting metabotropic receptors and/or by allowing calcium inflow into the cell (e.g., during LTP; Nelson et al., 2003; Lisman et al., 2012). Furthermore, through “asynchronous release,” even one presynaptic spike may be capable of eliciting prolonged release, and postsynaptic action, of fast neurotransmitters in vital brain circuits (Atasoy et al., 2012). How neuropeptides complement or facilitate such phenomena is unclear. Ample evidence, however, suggests that presynaptic signaling codes for the release of small molecule and neuropeptide transmitters may be different. This may expand the dynamic range of neuronal signaling by allowing small molecule transmitters to be released without presynaptically colocalized neuropeptides, and neuropeptides to maintain transmission when prolonged stimulation depletes the supply of fast transmitters.

## Synaptic vocabulary: different neural codes for different transmitters

Although the small diameter (<1 μm) of mammalian brain terminals makes it difficult to study this issue directly, the release of neuropeptides is thought to require a different type of axonal firing code than that of small-molecule transmitters. High-frequency firing, and/or burst firing, may be required to release the neuropeptides, whereas small-molecule transmitters require less intense electrical stimulation (Salio et al., 2006; Leng and Ludwig, 2008; Van Den Pol, 2012). This is thought to be at least in part related to different locations of the two types of transmitters within the presynaptic terminal: glutamate/GABA in small clear vesicles docked closely to the membrane, and peptides in large dense-core vesicles further away from the membrane (Salio et al., 2006; Leng and Ludwig, 2008). Intermediate-size neurotransmitters, such as catecholamines, have been reported to be located in both large dense-core vesicles (e.g., same vesicles as NPY, De Rijk et al., 1992) and small clear vesicles (El Mestikawy et al., 2011). Calcium is a key trigger for the release of both vesicles, and the near-membrane co-localization of calcium channels with the small vesicles, but not with large vesicles, is considered at least in part responsible for the observation that the amino acid transmitters are released by large local [calcium] elevations near the calcium channels (like those produced by an action potential), whereas neuropeptide release may require global [calcium] elevations in the terminal cytosol (Verhage et al., 1991; Van Den Pol, 2012). While the global [calcium] rise implicated in neuropeptide and catecholamine release may not have to reach the same high levels as those implicated in amino acid release (Verhage et al., 1991), the global [calcium] rise may require temporal summation of the effects of many action potentials. Indeed, at some central synapses, as many as several hundred spikes have been estimated to be needed on average to release a single neuropeptide vesicle (Leng and Ludwig, 2008), whereas the release of small-molecule transmitters such as glutamate can occur in response to a single spike. No broad “rules” linking specific firing rates with different types of transmitters have yet been formulated, except it has been consistently reported that higher frequencies and/or more prolonged stimulation are required to release “larger” transmitters than “smaller” transmitters. Recent examples include GABA (<1 Hz stimulation) and kisspeptin (5–10 Hz stimulation) release from anteroventral periventricular nucleus (Liu et al., 2011), and the release of glutamate (single-spike) vs. acetylcholine (>20 Hz prolonged stimulation) from habenula neurons (Ren et al., 2011).

What are the implications of these release requirements for neural processing? Considering that higher spike frequencies can be associated with higher failure rates of fast transmission (e.g., Schöne et al., 2012), do neuropeptides expand the dynamic range of synapses in firing ranges where the fast transmitter release saturates? This seems especially logical for slow behaviors such as sleep and feeding, which—unlike, for example, sensory processing—do not require millisecond-scale responsiveness to the environment, but may require prolonged neural activity better sustained by neuropeptides and/or intrinsically active neurons so often found in hypothalamic circuits (Burdakov, 2005).

Although many neuropeptides have direct postsynaptic effects on membrane excitability, many authors caution that this “should not perhaps be thought of as their main biological action” (Salio et al., 2006), and that a conceptual framework that includes “peptides as equal and independent transmitters should be replaced by one where peptides are viewed as essential and important modulators of GABA and glutamate actions” (Van Den Pol, 2003). Such views presumably stem from the well-documented presynaptic and/or non-electrophysiological (e.g., genomic) actions of neuropeptides. Indeed, recent optogenetic studies of peptidergic neurons, reviewed below, showed that these cells release fast transmitters, but have not yet revealed significant postsynaptic electrical effects of endogenously released neuropeptides. Specifically how this integrates into a coherent view of co-transmission is currently unclear, but current hypotheses envision neuropeptides as commitment signals functionally binding networks together over long time-scales, or in the words of Leng and Ludwig, “public announcements” vs. “whispered messages” delivered by fast and brief-acting neurotransmitters (Leng and Ludwig, 2008).

Overall, the material reviewed in this section, together with experiments highlighting the possibility of release of different transmitters from different parts of the same neuron (Fisher et al., 1988; Sossin et al., 1990; Sabatier et al., 2003), suggests that frameworks for functional dissection of co-transmission in a neuronal population should encompass at least three levels of analysis: chemical, electrical, and anatomical. Below, we review selected recent developments relating to these aspects of hcrt/orx circuits, which continue to captivate the authors' attention due to their key role in maintaining sleep–wake cycles and metabolic health in mammals.

## Intrinsic diversity within a cell type: example of hcrt/orx neurons

### Electrophysiological diversity of hcrt/orx circuits

Intrinsic firing “signatures” of neurons, which arise from different complements and/or distributions of ion channels they express, captivated neuroscientists for decades (Llinas, 1988; Mainen and Sejnowski, 1996), and more recent studies show that variation in intrinsic electrical properties within the same neuronal type can predict circuit and behavioral function (Angelo et al., 2012). When hcrt/orx neurons are examined with standard “electrical fingerprinting” protocols, such as membrane potential “rebound” after artificially imposed hyperpolarization, two distinct electrical classes emerge. “H type” hcrt/orx cells display hyperpolarized post-inhibitory rebound whereas “D type” hcrt/orx cells display depolarized post-inhibitory rebound, and the two cell types can be confirmed to be distinct by formal cluster analysis (Schöne et al., 2011). The two hcrt/orx cell types display distinct responses to physiological elevations in extracellular glucose levels: in H hcrt/orx cells, glucose evokes sustained hyperpolarization thereby allowing these cells to function as possible “glucostats” (sensors of absolute glucose levels), while D hcrt/orx cells show only temporary hyperpolarization and thus may act as adaptive sensors of trends in glucose levels (Williams et al., 2008). The two cell types also differ in fast synaptic inputs they receive, in particular the glutamatergic minis are larger and more frequent in H hcrt/orx cells (Schöne et al., 2011), and may be differentially affected in Huntington's disease, with H hcrt/orx cells experiencing an abnormally high glutamatergic drive (Williams et al., 2011).

### Projections and morphology of hcrt/orx neurons

The complexity and diversity of hcrt/orx neuron function is reflected in their widespread axonal projections throughout the brain, including key regions involved in the regulation of appetite, addiction, anxiety and wakefulness (Peyron et al., 1998). Attempts have been made to map these different functions to different anatomical subpopulations of hcrt/orx neurons. Activation of hcrt/orx neurons of the lateral parts (LH) of the hypothalamus has been implicated in reward related behaviors, whereas activation of hcrt/orx neurons in perifornical and dorsomedial parts (PFA-DMH) of the hypothalamus has been implicated in arousal (Harris and Aston-Jones, 2006). Indeed, topographically different subsets of hcrt/orx cells may be quite differently regulated by inputs (Fadel et al., 2002; Yoshida et al., 2006). While in the mouse, the biophysically distinct D and H hcrt/orx neurons are not exactly split between LH and PFA-DMH parts of the hypothalamus, the ratio of D to H type hcrt/orx neurons is higher in the PFA-DMH compared to the LH (Williams et al., 2008). However, in the mouse, the projections from LH and PFA-DMH hcrt/orx cells, and from H and D types of hcrt/orx cells, converge on both the locus coeruleus and ventral tegmental area (Gonzalez et al., 2012). This suggests, that at least in terms of those arousal (locus coeruleus) and reward (ventral tegmental area)-regulating regions, topographically similar hcrt/orx cells may control arousal and reward in the mouse (Gonzalez et al., 2012) and rat (Fadel et al., 2002; Espana et al., 2005).

Morphologically, H hcrt/orx cells have more complex dendrites than D hcrt/orx cells, at least in young animals (Schöne et al., 2011). However, in terms of number of dendric branch points and overall tree complexity, the dendritic structure of hcrt/orx cells is relatively simple compared to, for example, cortical and cerebellar projection neurons, and resembles the simple dendritic trees of other hypothalamic projections neurons and midbrain dopamine neurons (Grace and Onn, 1989; Armstrong, 1995; Stern, 2001; Schöne et al., 2011). Interestingly, since H hcrt/orx cells have more complex dendrites and stronger synapses than D hcrt/orx cells, and could thus comprise a preferential source of neural input to the hcrt/orx system (Schöne et al., 2011).

### Transmitters and receptors that decode electrical activity in hcrt/orx axons

The firing rate of hcrt/orx neurons is causally linked to awakening in a frequency-dependent manner, and this link is at least partly dependent on the expression of hcrt/orx (Adamantidis et al., 2007). However, apart from hcrt/orx peptides, the “hcrt/orx” neurons express a multitude of other transmitters and signaling molecules. These include the opioid peptide dynorphin (Chou et al., 2001), galanin (Hakansson et al., 1999), prolactin (Risold et al., 1999), and neuronal activity-regulated pentraxin (Reti et al., 2002; Crocker et al., 2005). Furthermore, different proportions of hcrt/orx cell bodies also have been found to express glutamatergic and GABAergic markers, which may suggests that not all hcrt/orx neurons express the same set of transmitters (Rosin et al., 2003; Torrealba et al., 2003; Harthoorn et al., 2005; Henny et al., 2010). Co-expressed transmitters could be contained in different vesicles or share the same vesicles. The later could lead to “vesicular synergy,” a novel mechanism where the presence of transporters for one transmitter enhances the function of transporters for the other transmitter, thereby increasing the vesicular transmitter content (El Mestikawy et al., 2011). Whether all transmitters expressed by a single hcrt/orx neuron all act at all its projection targets cannot be predicted from immunocytochemical or electron microscopy data available so far, in part because the axons and terminals of a single neuron are difficult to immuno-label and trace to postsynaptic partners (although not impossible, Rancz et al., 2011), and partly because a proof of “action” requires rapid functional assays at synaptic level (see next section).

The trafficking and/or synthesis of transmitters expressed in hcrt/orx may depend on target area and homeostatic state. This is indicated by maximum hcrt/orx levels in preoptic/anterior hypothalamus at the beginning of the light phase, but at the beginning of the dark phase in pons (Taheri et al., 2000). Levels of hcrt/orx in the cerebrospinal fluid levels are also highest at night (Fujiki et al., 2001), in line with maximal activity of hcrt/orx cells at this clocktime (Estabrooke et al., 2001; Kiyashchenko et al., 2002; Lee et al., 2005; Mileykovskiy et al., 2005). Similarly to hcrt/orx peptides, the levels of dynorphins are also regulated throughout the circadian rhythm, with levels increased during the active circadian phase or upon water deprivation (Przewlocki et al., 1983). Thus, the strength of different signaling molecules could vary differently depending on the projection target, circadian time, or body homeostasis, leading to corresponding changes in hcrt/orx cell signaling to postsynaptic targets. Important actions of non-hcrt/orx transmitters expressed in hcrt/orx neurons may explain why the feeding, body weight, and stress-induced thermogenesis abnormalities of hcrt/orx cell-ablated mice are more severe than those in hcrt/orx peptide-ablated mice (Hara et al., 2005; Zhang et al., 2010).

Since the effects of neurotransmitters expressed in hcrt/orx neurons are constrained by the availability of receptors, the distribution of receptors mediating hcrt/orx neuron signaling is key to understanding target-specific action of hcrt/orx neurons. The two known GPCRs for the hcrt/orx peptide show differential distribution, but partially overlap (reviewed in Sakurai, 2007). Dynorphin-containing fibers are found in most brain regions containing hcrt/orx fibers, but because there are many sources of dynorphin in the brain, it is currently unclear which of these fibers come from hcrt/orx neurons (Fallon and Leslie, 1986). Bath application of neuropeptides such as hcrt/orx or dynorphin revealed presynaptic effects on both GABAergic and glutamatergic synaptic transmission, as well as direct postsynaptic actions on membrane excitability of target cells (Van Den Pol et al., 1998; Burdakov, 2004). However, endogenously released transmitters might have different concentrations and time courses of action from those in artificial-application experiments. Especially, since hcrt/orx neurons express more than one transmitter, these will, when directly released from terminals together with other endogenous signaling molecules, have complex interactions and could activate multiple feedback mechanisms. There are currently no direct studies of this issue, but there are interesting data from artificial co-application of dynorphin and hcrt/orx at different postsynaptic targets, showing target-specific effects and different rates of desensitization for the two peptides (Li and Van Den Pol, 2006). Release of glutamate by hcrt/orx neurons, probed using optogenetics, is reviewed separately below.

### How do neurochemistry, neuroanatomy, and electrophysiology fit together?

**Figure 1:**
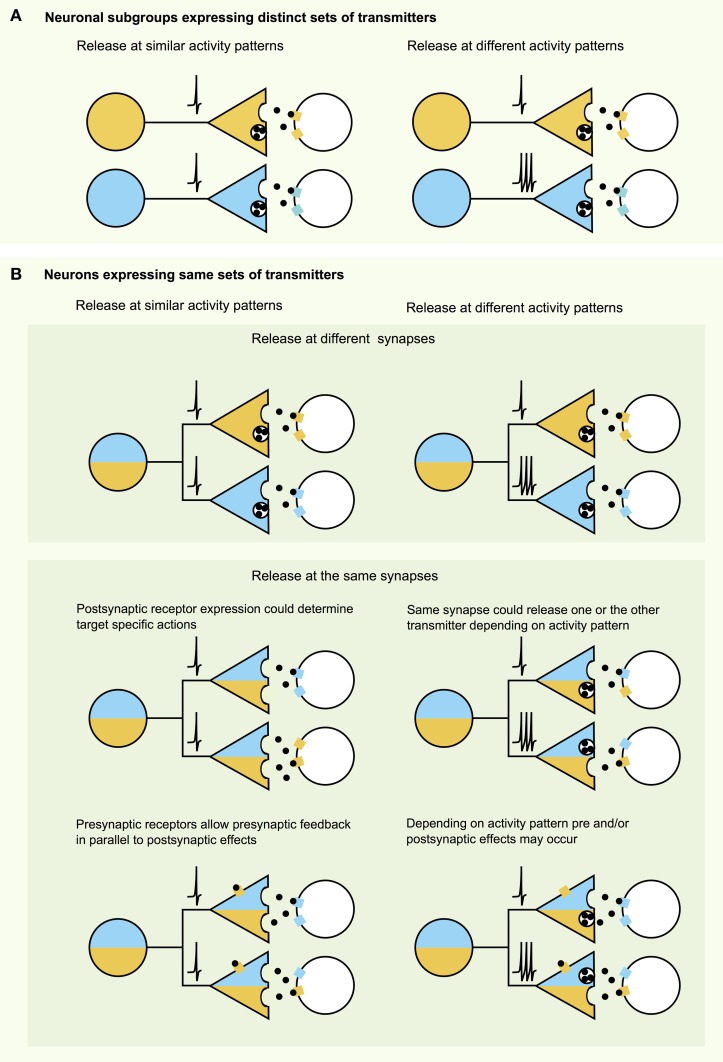
**Neurotransmission with multiple signaling molecules—a combinatorial analysis. (A)** When a certain cell type expresses multiple transmitters, these may act differentially on specific targets with subgroups of neurons expressing subsets of transmitters and modulating different target areas. **(B)** However, multiple transmitters expressed in the same cell still may affect their targets differentially. As illustrated in the top panel transmitters may be targeted to different release sites or may need different activity patterns (e.g., amino acid transmitters vs. neuropeptides). Middle panel: Even though transmitters may be released at the same sites, postsynaptic receptor expression or activity patterns may limit the action of one or the other transmitter on the postsynaptic target. Bottom panel: Transmission can be further altered and diversified by transmitter actions on presynaptic receptors.

How is signaling from endogenous hcrt/orx axons implemented on the synaptic level at different targets? Theoretically, there are many combinations of ways by which hcrt/orx neurons can influence their targets (Figure 1). Taken together, the high diversity in the hcrt/orx system may be the key feature to enable these neurons to differentially modulate their targets. These features may be: (1) At the target: Multiple receptors at distinct pre- or postsynaptic sites, with different affinities, and time-courses of action, and (2) At the release site: Subsets of hcrt/orx neurons expressing different sets of transmitters, projecting to different target areas, and distinct biophysical properties of these neurons, as well as differential targeting of transmitters to different regions of the brain at different times throughout the circadian rhythm to allow hcrt/orx neurons to regulate arousal and appetite according to homeostatic and environmental factors.

## Optogenetics reveals fast transmission from classical “slow modulatory” neurons

**Figure 2:**
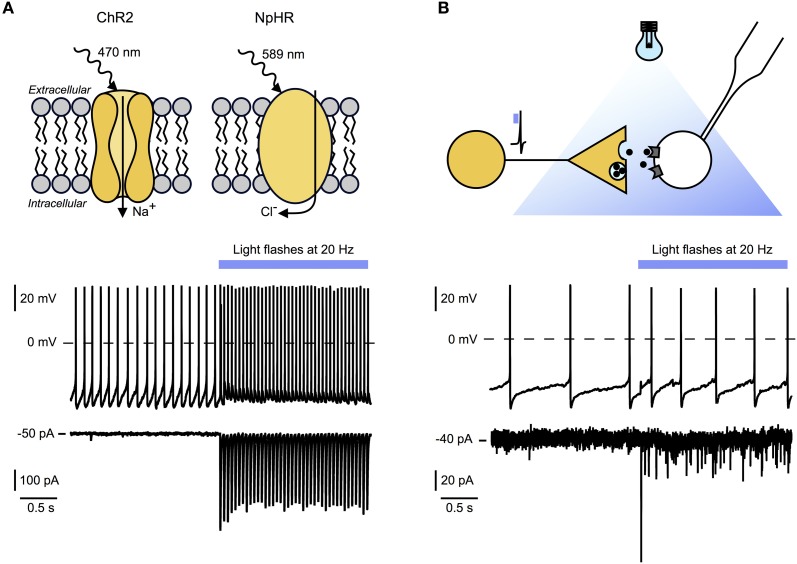
**Optogenetic tools for circuit mapping and functional characterization of synaptic signaling modes. (A)** Top panel: Example of light activated excitatory and inhibitory optogenetic tools. Channelrhodopsin 2 (ChR2) is a sodium channel sensitive to blue light. Targeted to specific cell types it causes fast (millisecond scale) depolarization and stimulation of action potentials in these cells. Halorhodopsin (NpHR) is a light activated chloride pump which causes hyperpolarization and suppression of action potential firing in target neurons. Bottom and middle panel: Whole-cell recordings of an hcrt/orx neuron expressing ChR2 before and during 20 Hz optical stimulation (blue bar). Bottom: Voltage clamp recording showing time locked currents evoked by blue light flashes. Middle: Current clamp recording with baseline firing rate and time locked action potential firing during optical stimulation. **(B)** Top panel: Experimental scheme illustrating experimental setup for ChR2-assisted circuit mapping. Yellow neuron, expressing ChR2, will release signaling molecules after optical stimulation. Time locked responses in postsynaptic cell (white) can be recorded using whole-cell patch-clamping. In combination with pharmacological blockade this technique allows the determination of receptors and transmitters involved. Bottom and middle panel: Recordings of a histamine neuron responding to 20 Hz optogenetic stimulation of hcrt/orx fibers (blue bar). Bottom: Voltage-clamp recording showing time locked postsynaptic currents evoked by transmitters release from hcrt/orx terminals in response to blue light flashes. Failures indicate that not all action potentials lead to vesicle release. Middle: Current-clamp recording with baseline firing rate and increased action potential firing during optical stimulation of hcrt/orx fibers.

At present, optogenetics is the only technique that allows causal links to be made between firing rates and transmitter release from genetically defined sets of axons inside native brain networks (Gradinaru et al., 2010; Bernstein et al., 2011; Miesenbock, 2011). The selectivity and power of optogenetics stems from genetic targeting of fast “light-switches” for neuronal activity, and from the non-invasive nature of light used to control those switches. Optogenetic tools can be used to manipulate the membrane potential of cells on a millisecond timescale. This approach uses light sensitive proteins such as excitatory channelrhodopsin-2 which is an ion channel permeable to sodium, or inhibitory chloride pump halorhodopsin (Figure 2A, top panel). There is now a growing repertoire of light-sensitive proteins with improved or novel functions, which are reviewed elsewhere (Yizhar et al., 2011). They then can be used in genetically identified neurons to directly stimulate or inhibit action potential firing in cell bodies or axons *in vivo* and *in vitro*.

Following the *in vivo* optogenetic demonstration that increases in hcrt/orx cell firing rate can cause awakening (Adamantidis et al., 2007), optogenetics has more recently been used to investigate the endogenous synaptic transmission from hcrt/orx neurons to their local-circuit partner, the histamine neurons of the tuberomammilary hypothalamus. In this study, channelrhodopsin-2 was targeted to hcrt/orx axons which were then depolarized with millisecond precision to recreate the firing rates recorded from hcrt/orx cells *in vivo*, while using membrane current and potential recordings from histamine neurons as an endogenous “bioassay” to sense transmitters released from hcrt/orx axons (Schöne et al., 2012). Stimulation of channelrhodopsin-containing fibers with millisecond flashes of excitation light generated fast postsynaptic currents in histamine neurons, with a high connection probability (Schöne et al., 2012) (Figure 2B, top and bottom panel). These currents were completely abolished by blockade of AMPA-type glutamate receptors. Furthermore, during brief (10 s) trains of optogenetically induced spikes in hcrt/orx axons at physiological firing frequencies, the glutamate inputs were necessary and sufficient to translate the electrical activity in hcrt/orx axons into increases in histamine cell firing (Schöne et al., 2012). This suggests that, at least under brief-stimulation conditions, the variations in electrical activity of endogenous hcrt/orx axons can induce glutamate-driven changes in electrical signals generated by histamine neurons without a requirement for other co-transmitters. It will be important to determine the role of these findings in *in vivo* observations of striking instability of consciousness caused by knockout of hcrt/orx peptides or receptors (Chemelli et al., 1999; Mieda et al., 2011), especially if this leaves the glutamate release from hcrt/orx cells unaltered.

Similar observations have recently been made during optogenetic probing of other neurons previously considered to communicate with their postsynaptic targets via the release of slow “modulatory” transmitters. For example, optogenetic stimulation of arcuate hypothalamic neurons expressing diverse neuropeptides derived from POMC leads to release and fast postsynaptic actions of either glutamate or GABA (Dicken et al., 2012). Optogenetic stimulations from neighboring feeding-promoting neurons expressing neuropeptides AgRP and NPY results in postsynaptic electrophysiological effects that are completely blocked by GABA antagonists in at least some postsynaptic targets, although both GABA and NPY signaling are necessary for the stimulation of AgRP cell axons to be translated into feeding (Atasoy et al., 2012). Classical “modulatory” neurons are no exception: when optogenetically stimulated, axons of mesolimbic dopamine neurons release glutamate in the nucleus accumbens (Tecuapetla et al., 2010). In turn, habenula cholinergic neurons release glutamate during brief optogenetic stimulation of their axons, and release acetylcholine during stronger and more prolonged stimulation (Ren et al., 2011).

Thus, recent evidence suggests that, at least at some of their synapses, neuropeptidergic, cholinergic, and aminergic neurons can use GABA or glutamate as primary fast neurotransmitters. How it relates to the action of classical modulatory transmitters they contain remains to be determined, but, considering that neuropeptides and other slow transmitters may only be released during intense activity, the ability of these neurons to release fast transmitters from small vesicles would certainly expand the dynamic range over which their firing can influence brain function.

## Overview: roles of co-transmission and neurotransmitter “identity crisis”

Powerful and fast (millisecond-scale) GABAergic and glutamatergic signaling from neurons previously considered to be primarily “modulatory” raises new questions about the roles of slower co-transmitters they co-express. The behavioral roles of this co-release remain poorly understood but are beginning to be unraveled by cell-specific knockout of selected neurotransmitters (Tong et al., 2007, 2008; Birgner et al., 2010; Alsio et al., 2011), although the results of such studies may be affected by developmental compensation in other neurotransmitters (e.g., knockout of NPY from NPY/AgRP neurons enhances their GABA transmission; Atasoy et al., 2012). Understanding circuit roles of co-expressed transmitters needs to include site-specific information about synaptic processing arrangements (Figure 1). This requires temporarily and spatially precise control over the activity of genetically specified synaptic terminals, which is now possible through optogenetics and/or modern spatial light patterning (Lutz et al., 2008).

A further confounding factor is the recent doubt shed on the idea that the neurotransmitter identity of a neuron remains the same after proliferation throughout the rest of a neurons' life. Activity-dependent re-specification of neurotransmitters has become an emerging topic in the homeostatic control of brain activity (Spitzer, 2012). Furthermore, the adult brain, and in particular the hypothalamus, may have the capacity to re-wire itself anatomically within hours (Horvath and Diano, 2004). Such rapid rearrangements in brain chemistry and morphology in response to experimental perturbations introduce uncertainty into the extent to which the natural “rules” of brain processing can be derived from slow and/or irreversible perturbations offered by classical genetics. Optogenetics offers fast and reversible ways of manipulating genetically defined pathways on the millisecond time-scales at which brain processes information. This could acheive greater certainty, especially when combined with rapid and direct “read-out” techniques such as classical electrophysiology. Such modern approaches may provide clearer answers about why it is advantageous for the brain to invest energy into making different classes of neurotransmitters and into segregating or combining them together.

### Conflict of interest statement

The authors declare that the research was conducted in the absence of any commercial or financial relationships that could be construed as a potential conflict of interest.
